# Comparison of microscopic adsorption characteristics of Zn(II), Pb(II), and Cu(II) on kaolinite

**DOI:** 10.1038/s41598-022-20238-z

**Published:** 2022-09-24

**Authors:** Li Tian, Kai-bin Fu, Shu Chen, Jun Yao, Liang Bian

**Affiliations:** 1 School of Environment and Resource, Southwest University of Science and Technology, Sichuan, 621010 People’s Republic of China; 2 Key Laboratory of Solid Waste Treatment and Resource Recycle, Ministry of Education, Southwest University of Science and Technology, Sichuan, 621010 People’s Republic of China; 3 School of Water Resources and Environment, China University of Geosciences, Beijing, 100083 People’s Republic of China

**Keywords:** Chemistry, Surface chemistry

## Abstract

In this research, kaolinite was used to investigate the comparative adsorption of copper, lead, and zinc ions through batch control experiments and first principles calculations. Different adsorption conditions were considered as the effect of solution acidity, initial concentration of ions, and contact shaking time. The adsorption system isotherms and kinetic studies were better agreed with the Langmuir and pseudo-second-order kinetic models. They reached adsorption equilibrium within two hours and maximum adsorption capacities of Zn(II), Pb(II), and Cu(II) on kaolinite were 15.515, 61.523, and 44.659 mg/g, respectively. In addition, the microscopic adsorption changes of Zn(II), Pb(II), and Cu(II) on kaolinite were characterized using X-ray diffraction, Fourier transform infrared spectroscopy, and scanning electron microscopy with energy dispersive X-ray spectroscopy. The results showed that Zn(II), Pb(II), and Cu(II) were most likely to be adsorbed on the kaolinite surface. Furthermore, the adsorption mechanism of [Zn(OH)]+, [Pb(OH)]+, and [Cu(OH)]+ on the kaolinite (001) surface was systematically studied through first-principles density functional calculations. The adsorption characteristics of different ions were evaluated by calculating the adsorption energy of the equilibrium adsorption configuration, state density, and electron density. The adsorption energy of [Zn(OH)]+, [Pb(OH)]+, and [Cu(OH)]+ were − 0.49,  − 1.17, and − 1.64 eV, respectively. The simulation results indicated that new hybrid orbitals were formed between the metal ions and O atoms on the kaolinite surface, with electron transfer occurring the adsorption processes. The charge transfer direction for [Pb(OH)]+ was opposite those for [Zn(OH)]+ and [Cu(OH)]+. [Zn(OH)]+ was more likely to form polydentate complexes with hydroxyl groups on the kaolinite surface than [Cu(OH)]+ and [Pb(OH)]+. This work further elucidated the interaction mechanism between the adsorption systems and provided fundamental theoretical support for the structural modification and optimization of kaolinite, such as increasing the layer spacing of kaolinite and introducing other active groups on its surface to improve the adsorption capacity of heavy metal ions in water treatment and soil remediation.

## Introduction

Rapid industrialization across the globe in recent years has led to the widespread emissions of, heavy metals into the natural environment through various industrial processes, such as electroplating, tanning, alloying, fertilizer production, papermaking, and pesticide production. Heavy metals are one of the main causes of water and soil pollution^1^. Due to the high toxicity of heavy metals, water-based organisms are ineffective at degrading large amounts of heavy metals, and even interact with some heavy metals to convert them into more toxic substances that cause serious harm to the ecosystem. In addition, heavy metals in the food chain, especially due to polluted water bodies, which directly threatens human living environments and may indirectly cause several serious health problems, such as brain injury, anemia, infertility, and liver and kidney diseases^2–5^. Therefore, once heavy metals are discharged into the water body, it is difficult to remove them completely. Zn(II), Pb(II), and Cu(II) are typical heavy metal pollutants, and can cause considerable damage to biological systems ^6,7^. Adsorption is a promising method for treating water and soil containing heavy metal ions^8^. Compared with chemical precipitation, ion exchange, and electrolysis methods, adsorption is simpler to operate, more efficient, and produces fewer secondary pollutants. It is widely used in the removal or stabilization of pollutants in the ecological environment^9,10^.

Consequently, a large number of studies are underway in various countries to discover low-cost and environmentally friendly materials for the removal of heavy metal pollutants from the aquatic environments^11^. Clay minerals have attracted much attention in recent years because of their availability, stable structure, and low price^12,13^. Numerous studies have proved that natural clay minerals and their modified products have unique physical and chemical properties, such as biocompatibility, non-toxicity, and antimicrobial activity, making them effective materials for removing various pollutants from aqueous media^14,15^. Due to the high content of O-containing functional groups in clay minerals, chemical interactions and physical synergistic effects related to the pollutants are easily produced on the surface of clay minerals. However, the removal efficiency of adsorption technology is closely related to the interaction mechanism between the adsorbents and adsorbates^16,17^. The main factors affecting the interaction mechanism are the physicochemical and structural properties of the reactants at the macroscopic level and their electronic structures at the microscopic level^17,18^. Kaolinite is a natural clay mineral composed of Si tetrahedrons and Al or Mg octahedrons. It is a 1:1 layered silicate^19^. Due to the unique spatial structure of kaolinite and large specific surface area, it is widely used to treat heavy metals, and there have been several micro- and macro-level studies on this subject. Iannicelli-Zubiani et al. studied the use of natural clays as sorbent materials for rare earth ions, and, as part of this study, they characterized the material and established the parameters^20^. Zhang et al.^21^ studied the intercalation modification mechanism of kaolinite and found that the adsorption capacity was greatly increased. Wang et al.^22^ studied the adsorption of Zn(II) on the surface of kaolinite (001). In this work, the bonding mechanisms between Zn(II), Pb(II), Cu(II), and O-containing functional groups on the surface of kaolinite were explored through batch experiments and simulation calculations, and important factors affecting the adsorption rates, such as pH, initial concentration, adsorption time, and temperature, are discussed. The adsorption mechanism was studied through characterization analysis, density functional theory (DFT) calculations, and differential charge density analysis. DFT calculations can be used to determine the energy, structure, and electronic properties of atoms and molecules, which can enable interpretation at the atomic level^23,24^. The results of this work will contribute to the understanding of the adsorption mechanism of heavy metals in different clay minerals and provide a new direction for clay mineral modification.

## Materials and methods

### Experiment materials

The kaolinite was purchased from Chengdu Chenzheng Chemical Co., Ltd. Its X-ray fluorescence results showed that, the main components of kaolinite are SiO_2_ (55.2%), Al_2_O_3_ (38.74%) and K_2_O(3.27%). Zn(NO_3_)_2_, Pb(NO_3_)_2_, and Cu(NO_3_)_2_ were purchased from Sinopharm Chemical Reagent Co. Ltd. They were of analytical grade and were used without further purification. Deionized water was used for the preparation of all solutions. The pH adjustment of solutions was performed using 0.1 mol/L HNO_3_ or 0.1 mol/L NaOH.

### Adsorption experiment and detection methods

Stock solutions of Zn(II), Pb(II), and Cu(II) were prepared by dissolving Zn(NO_3_)_2_, Pb(NO_3_)_2_, and Cu(NO_3_)_2_, respectively, into deionized water and diluting to 1000 mg/L. The adsorption performance of kaolinite was examined using batch mode studies, and duplicate experiments were also performed. Adsorption kinetics studies were conducted at ambient temperature (298 ± 2 K) for 300 min in 250 mL conical flasks containing 100 mL of the 300 mg/L metal solutions and 300 mg kaolinite. The adsorption isotherms of Zn(II), Pb(II), and Cu(II) on kaolinite (300.0 mg) were investigated by performing adsorption with initial concentrations of 50–300 mg/L, followed by constant shaking at 165 rpm, pH = 5.0 ± 0.1, and 298 ± 2 K for 6 h. In addition, the effect of solution pH (2–7) was observed for Zn(II), Pb(II), and Cu(II) adsorption. After the adsorption test, the supernatants were filtrated using a 0.45 μm pore size filter, and the residual concentrations of Zn(II), Pb(II), and Cu(II) were detected using inductively coupled plasma optical emission spectrometry. The adsorption capacities (q_e_, mg/g) of kaolinite for Zn(II), Pb(II), and Cu(II) were estimated from mass balance calculations according to Eq. (1).

1 $${\text{q}}_{{\text{e}}} = \frac{{\left( {{\text{C}}_{0} - {\text{C}}_{{\text{e}}} } \right){\text{V}}}}{{\text{m}}}$$

where C_0_ and C_e_ (mg/L) are the initial and equilibrium concentrations, respectively, of Zn(II), Pb(II), and Cu(II) in the measured solution; V (L) is the volume of the measured solution; and m (g) is the mass of the added kaolinite.

### Characterization methods

Surface area analyzer (ASAP 2460 Version 3.01) was applied to determine the specific surface area of kaolinite. Besides, the surface topography, layer spacing, and bonds changes of kaolinite before and after adsorption were investigated scanning electron microscopy with energy-dispersive X-ray spectroscopy (SEM–EDS), X-ray diffraction (XRD), and Fourier-transform infrared (FT-IR) spectroscopy.

#### SEM–EDS

A small amount of dried kaolinite was fixed on the sample table with conductive double-sided adhesive, and then treated with gold spraying for 30 s. The surface morphology of the sample was observed by TM-1000 transmission electron microscope produced by HITACHI.

#### XRD

In this paper, X-ray diffractometer Axios advanced made by PANalyticalB.V was used for XRD test. The specific test conditions of the sample are as follows: Cu Ka target (λ = 1.5406 Å), photocell working voltage is 40 kV, working current is 40 mA, scanning Angle (2θ) is 4–70°, scanning step length is 0.5°, scanning speed is 0.1 s/step.

#### FT-IR

The surface groups of kaolinite were analyzed by FT-IR (Nicolet 5700). An appropriate amount of kaolinite was ground to powder, and then mixed with KBr. The mixture was pressed using a solid tablet press, and blank KBr was used as the background. The recorded spectra ranged from 4000 to 400 cm^−1^ with a resolution of 2 cm^−1^.

### Theoretical calculation methods

The crystal structure of the kaolinite cell was constructed based on the parameters obtained from previous studies on kaolinite performed using low-temperature (1.5 K) neutron powder diffraction. The kaolinite crystal structure file was imported to Materials Studio 8.0 (MS 8.0). The Cambridge Sequential Total Energy Package (CASTEP) was used to optimize the geometry and energy of the crystal structure. Previous studies have suggested that the (001) surface of kaolinite is mainly involved in adsorption, which consists of hydrophilic Al–O octahedrons^25,26^. Thus, on the basis of kaolinite bulk, a (001) surface section was carried out, and the cell chamber was expanded to establish a vacuum layer with a height of 18 Å to eliminate the periodic effects of different layers between adjacent slabs^27,28^.

To ensure the feasibility of the adsorption simulation, before simulation, the Visual MINTEQ software was used to calculate the existing states of Zn(NO_3_)_2_, Pb(NO_3_)_2_, and Cu(NO_3_)_2_ separately at a concentration of 100 m g/L, pH = 5, and 298 K in the aqueous solution. The existing states of their possible adsorptions were analyzed. The calculations results showed that the states of Zn, Pb, and Cu that may be adsorbed by kaolinite in aqueous solution are Zn(II), Pb(II), and Cu(II), respectively, and their respective complex ions, namely, [Zn(OH)]+ , [Pb(OH)]+ , and [Cu(OH)]+ . Because complex ion clusters are large and are more easily adsorbed, in this work,simple complex ions [Zn(OH)]+ , [Pb(OH)]+ , and [Cu(OH)]+ were used as examples to study the mechanism of adsorption on kaolinite (001).

**Figure 1:**
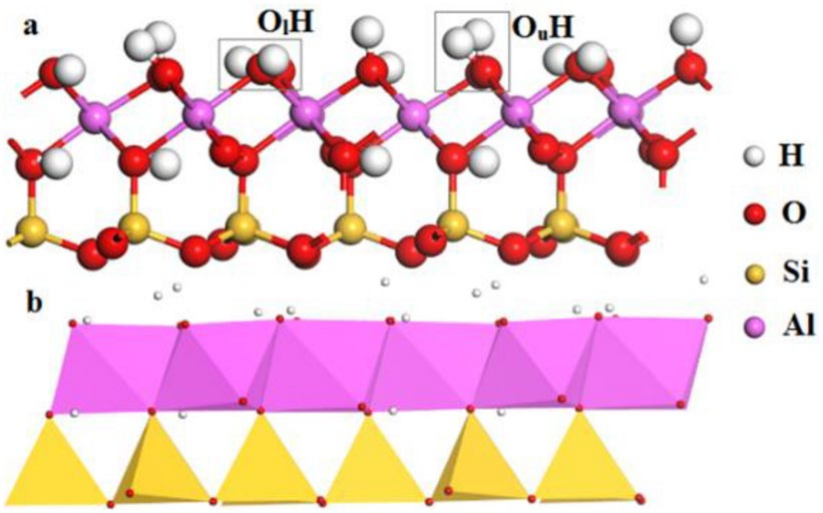
Structure of kaolinite (**a**): ball and stick model; (**b**): polyhedron model.

All simulations were performed using the CASTEP module in MS 8.0 with the Perdew Burke Ernzerhof functional based on the generalized gradient approximation, because of its high computational accuracy and efficiency in studying the surface process and, the properties of small molecules, H-bonded crystals, and crystals in the inner space^29^. A cutoff energy of 340 eV was adopted throughout and the max force and displacement were set at 0.1 eV/Å and 0.005 Å, respectively. To optimize the atom positions, a 1 × 1 × 1 grid K-point was sampled until the total energy changed. A higher cutoff energy or more refined K-point mesh only caused negligible changes in the results^30,31^. Before the simulation calculations, the models of [Zn(OH)]+, [Pb(OH)]+ and [Cu(OH)]+ were optimized under the same conditions to find their lowest energy states (stable configurations). After optimization, the Zn–O, Pb–O, and Cu–O bond lengths were 2.074, 3.301, and 1.815 Å, respectively. The structures of kaolinite and the (001) slab are shown in Fig. 1. The figure shows a clay mineral with a layered structure; the unit structure is a triclinic cell of the C1 space group, and is composed of alternating Al–O octahedrons and Si–O tetrahedrons^32^. In contrast to the kaolinite bulk, two types of surface hydroxyls were observed in the (001) slab. One third of the hydroxyls were “lying” hydroxyls (O_l_H) oriented parallel to the kaolinite (001) surface and the remaining two thirds were “upright” hydroxyls (O_u_H) oriented perpendicular to the surface^33^. Heavy metal ions bind with the O atoms of the Al–O bands on the surface of kaolinite (001) more strongly. When the metal ions bind to the O atoms, they become closer to the Al atoms. Thus, selected the position (x: 0.75, y: 0.36, z: 0.19) directly above the surface O_u_ (following article is denoted by O) of kaolinite to place [Zn(OH)]+, [Pb(OH)]+ and [Cu(OH)]+, respectively. Previous studies have shown that the force range of heavy metal atoms on O atoms is ~ 3 Å. Therefore, [Zn(OH)]+, [Pb(OH)]+, and [Cu(OH)]+ were respectively placed ~ 3 Å above the O atoms on the kaolinite (001) surface.

## Results and discussion

### Adsorption results and discussion

#### Zn(II), Pb(II), and Cu(II) adsorption performance

**Figure 2:**
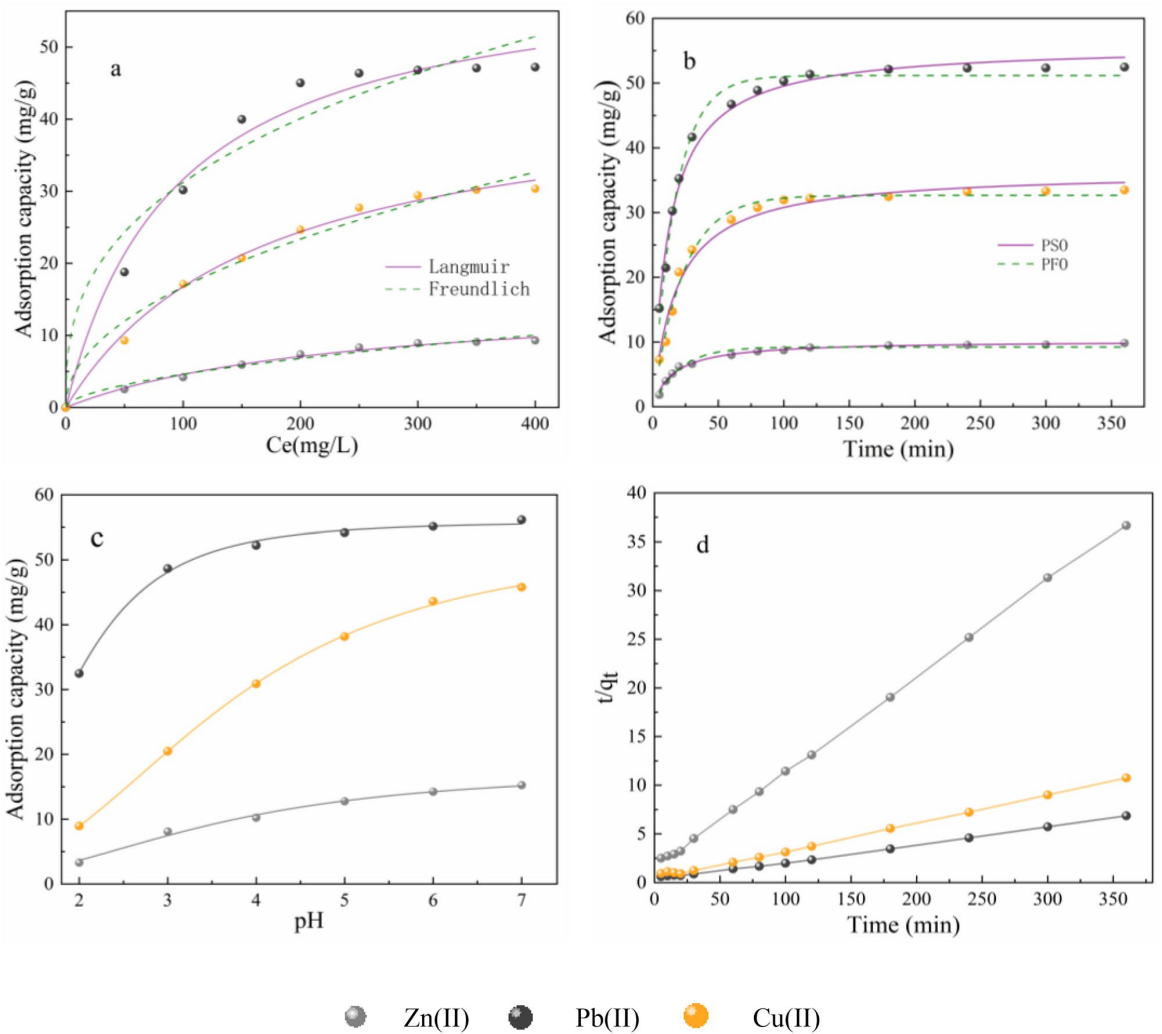
Adsorption isotherms of Zn(II), Pb(II), and Cu(II) on kaolinite (**a**). The effect of shaking adsorption time and PSO kinetic models of Zn(II), Pb(II), and Cu(II) adsorption on kaolinite (**b**, **d**). Effect of solution pH on the adsorption of Zn(II), Pb(II), and Cu(II) on kaolinite (**c**).

To explore the mechanism of removal of Zn(II), Pb(II), and Cu(II) using kaolinite, the influence of initial concentration on the removal capacities was examined. As shown in Fig. 2a, the removal capacities increased with increasing initial concentration, and kaolinite exhibited much higher adsorption affinity for Pb(II) than for Zn(II) and Cu(II). The electronegativity of heavy metals and the precipitated products on the surface are the key factors affecting their adsorption capacity on kaolinite. For Cu(II) and Zn (II), Cu(II) is more electronegative; for Pb(II), Cu(II), and Zn (II), Pb(II) is easier to precipitate due to its larger specific gravity. The adsorption capacity was fitted using Langmuir models and Freundlich models, and the fitting parameters are shown in Table 1. The isotherms results were better fitted Langmuir models than Freundlich models. The results showed that the maximum adsorption capacities of kaolinite for Zn(II), Pb(II) and Cu(II) were 15.515, 61.523, and 44.659 mg/g, respectively, which is in agreement with the experimental results. The adsorption capacity of the kaolinite material discussed herein is smaller than those of previously reported clay materials. Although the experimental conditions were slightly different, the clay materials still exhibited notable adsorption capacities for the remediation of heavy metal contaminated water.

**Table 1:** Fitted parameters summary of adsorption isotherm.

Adsorbate	Langmuir model			Freundlich model		
	Q_m_ (mg g^−1^)	K_L_ (L mg^−1^)	R^2^	n	K_F_ (mg g^−1^)/(mg L^−1^)^n^	R^2^
Zn(II)	15.515	0.004	0.990	0.560	0.350	0.974
Pb(II)	61.523	0.011	0.982	0.361	5.934	0.947
Cu(II)	44.659	0.006	0.995	0.483	1.812	0.976

The relation between reaction time and the adsorption of Zn(II), Pb(II) and Cu(II) at pH = 5.0 is shown in Fig. 2b,d. The reaction rate and adsorption capacity for Pb(II) were higher than those for Zn(II) and Cu(II), indicating that kaolinite could provide more adsorption sites for Pb(II). The adsorption time of Zn(II), Pb(II), and Cu(II) on kaolinite was short, with equilibrium being reached within 2 h. Pseudo-first-order (PFO) and Pseudo-second-order (PSO) kinetics modelling (Fig. 2b) were conducted to evaluate the adsorption mechanism of Zn(II), Pb(II), and Cu(II), and the fitting parameters are shown in Table 2. The kinetics results were better fitted PSO model than PFO, indicating the chemisorption plays a leading role in the reaction. The effect of shaking adsorption time and adsorption capacity of Zn(II), Pb(II), and Cu(II) on kaolinite are shown in Fig. 2d.

**Table 2:** The model parameters of adsorption kinetics.

Adsorbate	PFO model			PSO model		
	K_1_ (min^−1^)	Q (mg g^−1^)	R^2^	K_2_ (mg (g min)^−1^)	q (mg g^−1^)	R^2^
Zn(II)	0.049	9.221	0.967	0.006	10.214	0.976
Pb(II)	0.058	51.163	0.987	0.001	55.903	0.986
Cu(II)	0.043	32.660	0.988	0.001	36.390	0.989

To further investigate the influence of pH on the simultaneous removal efficiency for Zn, Pb, and Cu ions, the experiment was carried out in the 2.0–7.0 pH range (Fig. 2c). Cu(II) and Pb(II) began to deposit around pH 4.6 and 6.0, which affected the normal adsorption, and ions precipitation played an important role. Consequently, the removal efficiency of kaolinite for Pb(II) and Cu(II) were higher than Zn(II). The removal efficiency for Zn(II), Pb(II), and Cu(II) using kaolinite improved with increasing initial pH, and kaolinite showed the most significant removal ability for Pb ions in simultaneous removal from solution. These results provide a research basis for future engineering applications.

#### Characterization results and discussion

**Figure 3:**
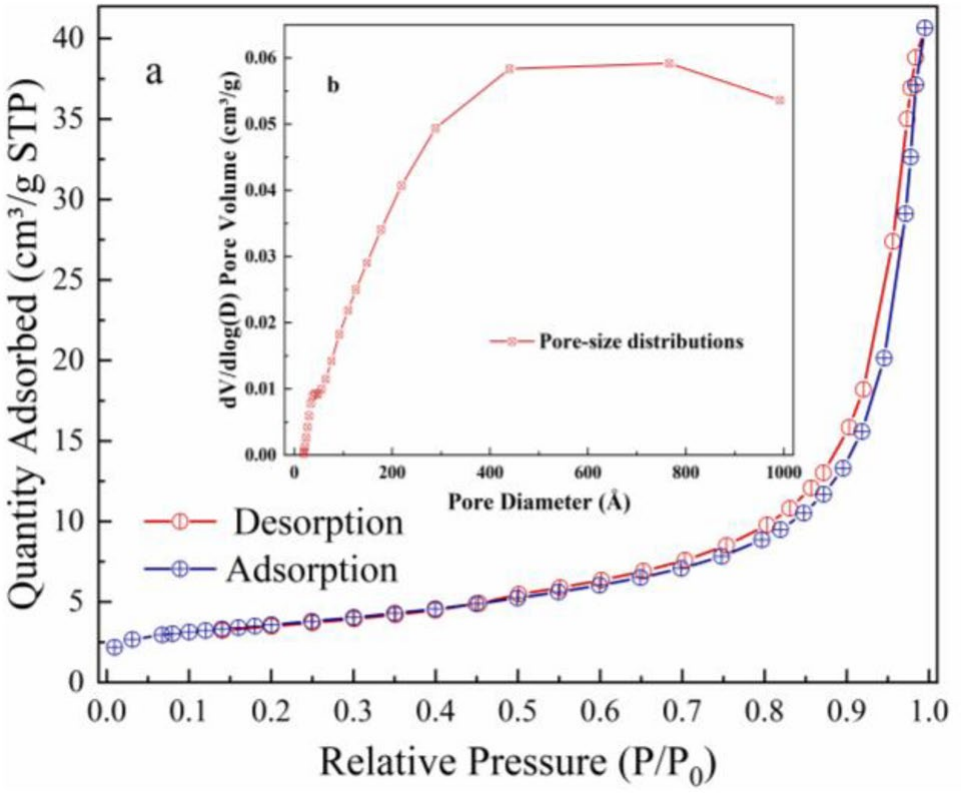
N_2_ adsorption–desorption isotherms (**a**) and pore-size distributions of kaolinite (**b**).

Figure 3a shows the N_2_ adsorption–desorption isotherms of the kaolinite. The sample showed a type IV isotherm, suggesting the presence of mesopores. In the relative pressure range of 0.3–1.0, this sample exhibited a H3 typehysteresis loop that could be attributed to its lamellar structure. Moreover, the BET surface are 12.57 m^2^/g. Non-metallic minerals with a large BET surface area was beneficial to immobilize heavy metals in soil and water. Corresponding pore-size distributions of kaolinite was shown in Fig. 3b. The pore diameter and total pore volume were 200.237 Å and 0.057 cm^3^/g.

**Figure 4:**
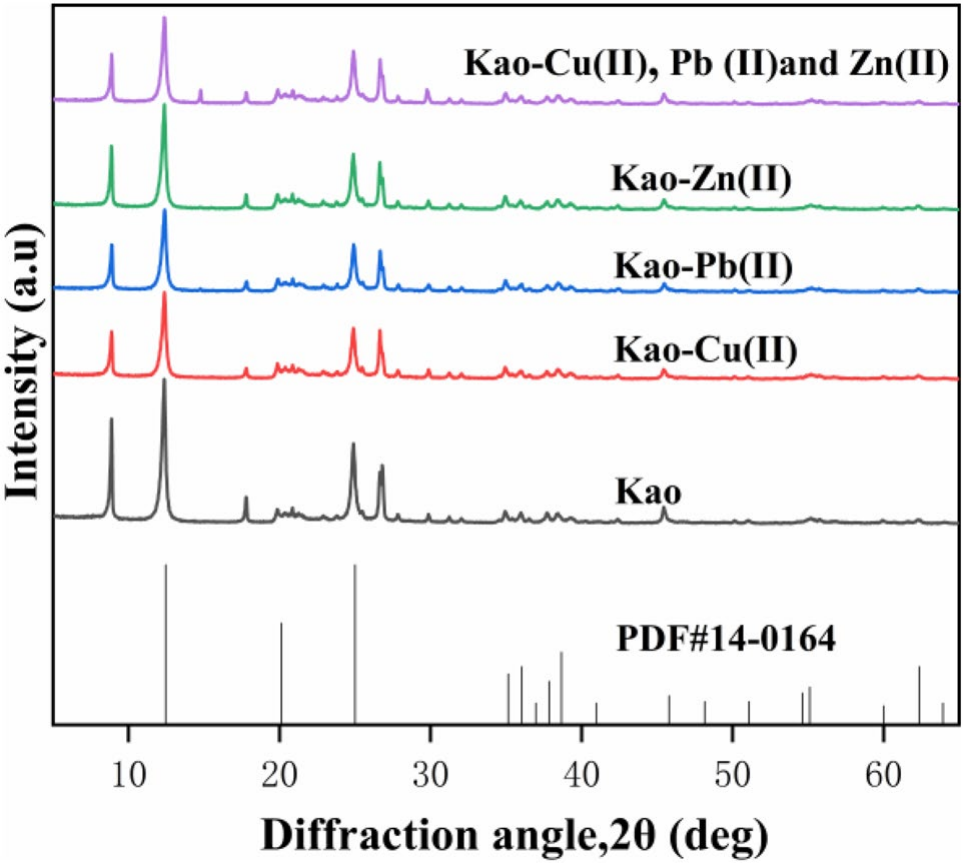
XRD patterns of kaolinite (Kao) before and after absorption of Zn(II), Pb(II), and Cu(II).

The XRD patterns of kaolinite before and after adsorption of Zn(II), Pb(II) and Cu(II) ions are shown in Fig. 4. The kaolinite characteristic pattern agreed with the standard ICDD reference patterns for kaolinite (PDF No. 14-0164). Kaolinite exhibited two characteristic crystalline peaks at 12.33° and 24.86° belonging to its (001) and (002) planes, which reveals its crystalline nature. The diffractogram of kaolinite showed a sharp (001) peak at 12.31°, which is consistent with previous reports^34,35^. After the adsorption of Zn(II), Pb(II), and Cu(II) ions, all the diffraction peaks were still clearly identified and no substantial differences were observed in the XRD pattern, indicating that the adsorption process did not affect the crystal structure of kaolinite. However, compared with the original kaolinite, the characteristic peaks of kaolinite after Zn(II), Pb(II), and Cu(II) adsorption were significantly weakened, indicating that the crystal morphology of kaolinite is not obvious after adsorption of metal ions on the surface. The interactions between structural units in kaolinite were mainly dominated by H-bonds. Zn(II), Pb(II), and Cu(II) ions do not have adequate capacity to break these H-bonds^36^; therefore, metal ions could rarely enter the inter layer spaces. Besides, kaolinite is known to have negligible isomorphic substitution^37,38^. Thus, the adsorption performance of kaolinite can be improved through intercalation to increase the interval between layers. The intercalation of kaolinite would increase the basal spacing of the 1:1 alumina/silicate layers, resulting in a shift of in (001) diffraction peak to a smaller angle^21,39^. The diffraction peak offset angle of kaolinite in Fig. 4 is very small, indicating that the kaolinite interlayer was not damaged. These results suggest that fewer Zn(II), Pb(II), and Cu(II) ions entered the interlayers and a majority of them were adsorbed on the surface of kaolinite.

**Figure 5:**
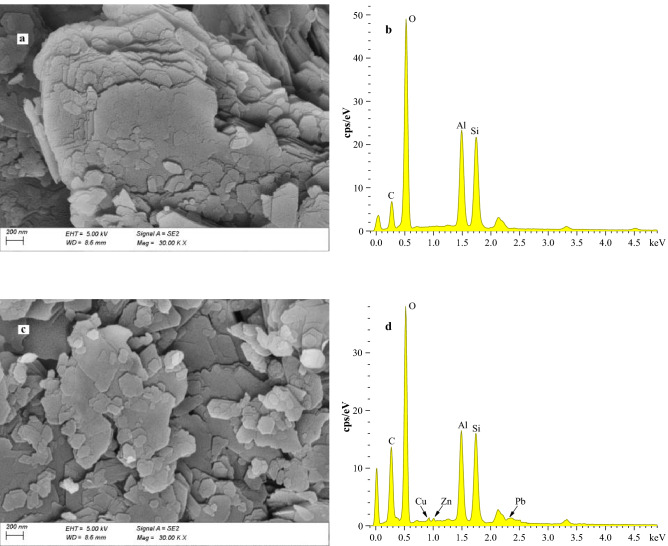
SEM–EDS images of kaolinite before (**a**, **b**) and after adsorption of Zn(II), Pb(II), and Cu(II) (**c**, **d**).

SEM–EDS was used to examine the morphology change before and after adsorption of clay minerals. As shown in Fig. 5a,c, the morphology change was very small. However, after treatment with a Zn(II), Pb(II), and Cu(II) ion solution, Zn(II), Pb(II), and Cu(II) peaks were observed (Fig. 5b,d), confirming the presence of cations on the kaolinite surface. The peaks of Pb(II) and Cu(II) were higher than that of Zn(II), which may be because Pb(II) and Cu(II) bind more readily with the groups on the kaolinite surface. The above analysis confirmed that Zn(II), Pb(II), and Cu(II) ions could interact with clay mineral surface sites, which was in agreement with the batch experiment results.

**Figure 6:**
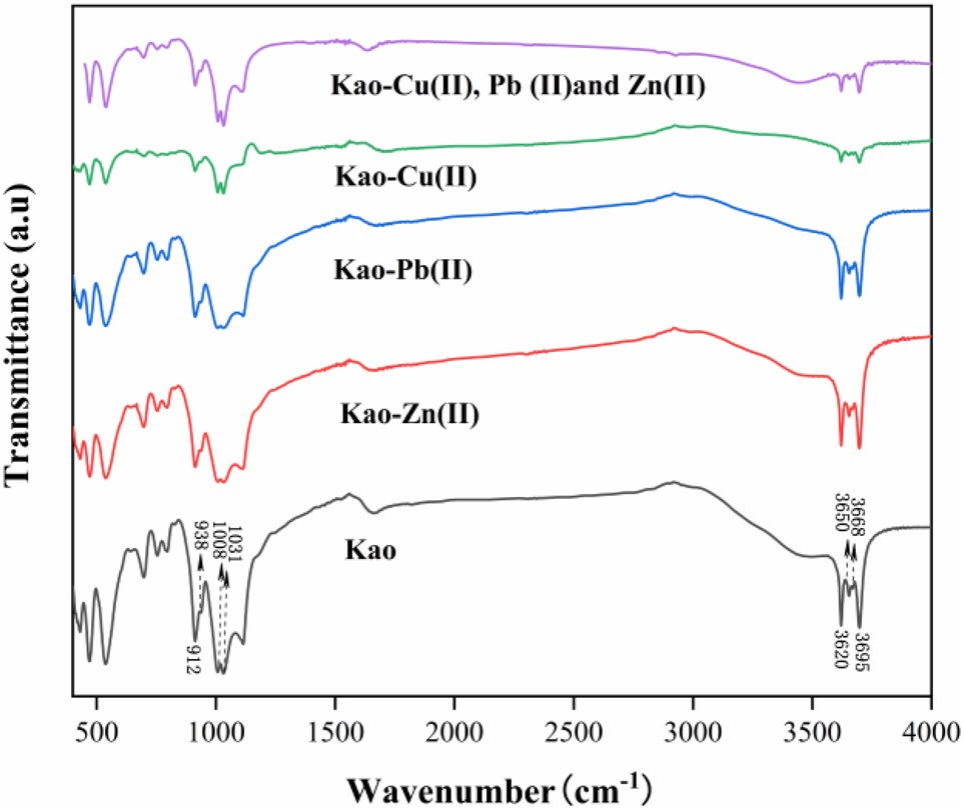
FT-IR spectra of kaolinite before and after absorption of Zn(II), Pb(II), and Cu(II).

FT-IR spectra of pristine kaolinite and kaolinite with adsorbed metals are shown in Fig. 6. The bands at 3695, 3668, and 3650 cm^−1^ in the spectrum of pristine kaolinite are attributable to stretching vibrations of the surface –OH groups exposed to the surface of the alumina interlayer^40^. The band at 3620 cm^−1^ is ascribed to the stretching vibration of –OH groups located within the kaolinite framework. The surface –OH groups can lose or gain protons in different media and are actively involved in the formation of H-bonds as H-donors and/or acceptors, whereas the internal –OH groups do not interact with intercalation compounds easily because they are recessed within the kaolinite structure. The intensity of the bands at 3695, 3668, and 3650 cm^−1^ changed upon Zn(II), Pb(II), and Cu(II) adsorption, implying that the surface –OH groups formed H-bonds with Zn(II), Pb(II) and Cu(II)^41^. Notably, after kaolinite adsorbed Cu(II), its stretching vibration peak was more prominent than those of Zn(II) and Pb(II), especially in the low-frequency region. This may be because surface ^−^OH groups easily form complexes with Cu(II) because the frequency of the ^−^OH stretching vibration is closely related to the geometric configuration of the complex. The bands at 1031 and 1008 cm^−1^ are attributed to Si–O stretching vibrations and while those at 938 and 912 cm^−1^ are caused by Al–O–H formation vibrations^42,43^. Figure 6 shows that the Si–O bond changes little before and after adsorption, indicating that it has low activity. The appearance of new characteristic peaks in the infrared spectrum indicates that mainly chemical adsorption occurs, as physical adsorption can only shift the characteristic absorption bands of adsorbed molecules or change the band intensity without generating new bands. Therefore, within the low-frequency regions, a new peak at about 1138 cm^−1^ emerged after kaolinite absorbed Cu(II), which means the adsorption was dominated by chemical reactions and a complex was formed. The interfacial –OH groups of kaolinite can undergo surface coordination complexation with anions, metal cations, and radicals in solution because of their amphiphilic nature, leading to the chemical adsorption shown in Eqs. (2) and (3). The surface of kaolinite has surface potential and surface charge, which imparts certain electrostatic characteristics and enables it to react with some ions in solution. On the whole, these peaks demonstrate that the basic framework structure of kaolinite was not destroyed by Zn(II) and Pb(II) and the original system remained. This results is consistent with the XRD finding that Zn(II), Pb(II), and Cu(II) did not enter the interlayers of kaolinite.

2 $${\text{ J}} - {\text{OH + R}}^{{{\text{n}} - }} {\text{ = J - OR}}^{{{\text{(n}} - {1)} - }} {\text{ + OH}}^{ - }$$

3 $${\text{ J}} - {\text{OH + M}}^{{\text{n + }}} {\text{ = J}} - {\text{OM}}^{{{\text{(n}} - {1) + }}} {\text{ + H}}^{ + }$$

where J is the kaolinite surface, R^n−^ is the weak acid ions or anionic ligands in the solution, M^n+^ is the metal ions in the solution.

### Simulation results and discussion

#### Optimized model parameters

Table 3 compares the optimized kaolinite volume unit cell parameters with the experimentally obtained parameters. The calculated unit cell parameters can better simulate the experimental parameters than the original crystal structure.

**Table 3:** The model parameters of kaolinite for adsorption.

Lattice parameters	Length (Å)			Angle (°)		
	a	b	c	α	β	γ
original	5.148994	8.933998	7.3840	91.9300	105.04196	89.7910
calculated	5.139840	8.915788	7.4822	91.3503	104.30327	89.9046

#### Equilibrium configuration and adsorption energy

After optimizing the initial model structures, the corresponding adsorption energy was calculated using the surface adsorption formula. The adsorption binding energy E_ads_ was calculated according to Eq. (4).

4 $${\text{E}}_{{{\text{ads}}}} = {\text{E}}_{{{\text{total}}}} { } - {\text{E}}_{{{\text{adsorbate}}}} - {\text{E}}_{{{\text{adsorbent}}}} { }$$

where E_total_, E_adsorbate_ and E_adsorbent_ represent the total energy of the system, adsorbate, and adsorbent, respectively.

**Figure 7:**
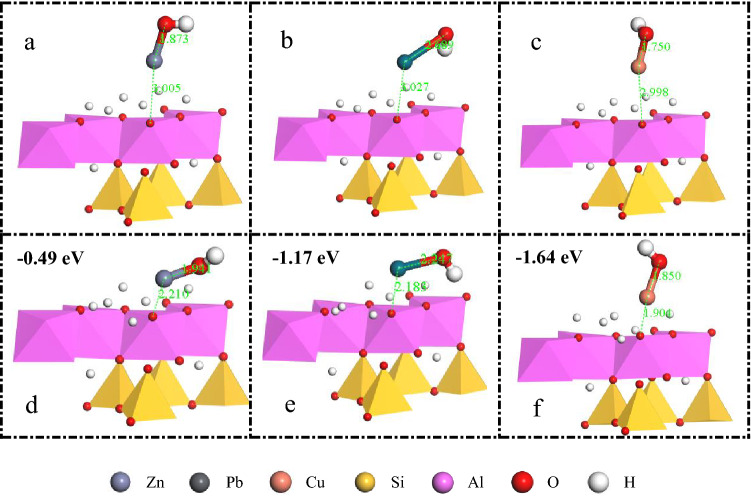
Initial adsorption configuration of [Zn(OH)]+, [Pb(OH)]+, and [Cu(OH)]+ on the surface of kaolinite (001) (**a**, **b**, **c**); Adsorption equilibrium configurations and binding energies of [Zn(OH)]+, [Pb(OH)]+, and [Cu(OH)]+ on the surface of kaolinite (001) (**d**, **e**, **f**).

The initial configurations of the [Zn(OH)]+, [Pb(OH)]+, and [Cu(OH)]+ adsorbed on kaolinite are shown in Fig. 7a–c. The adsorption energy, calculated using Eq. (4), and adsorption equilibrium configuration of the three substances are shown in Fig. 7d–f. The surface adsorption energy calculations showed that [Zn(OH)]+, [Pb(OH)]+, and [Cu(OH)]+ all had surface adsorption energies of less than 0, suggesting that an exothermic reaction occurs on the O surface of kaolinite, this indicates that the surface adsorption of these heavy metal ions on kaolinite is feasible. Further, a lower binding energy indicates a stronger adsorption binding affinity^44^. Comparing the adsorption configurations of kaolinite before and after adsorption, it is evident from the equilibrium adsorption configuration that [Zn(OH)]+, [Pb(OH)]+, and [Cu(OH)]+ all approach the surface of kaolinite to some extent and do not enter the interlayers of kaolinite, which is consistent with the XRD test results. The Zn-OH, Pb-OH, and Cu-OH bond lengths of [Zn(OH)]+, [Pb(OH)]+, and [Cu(OH)]+, respectively, also changed. The Zn-OH, Pb-OH, and Cu-OH bond lengths were stretched from 1.873 to 1.941 Å, compressed from 2.689 to 2.247 Å, and stretched from 1.750 to 1.850 Å, respectively. The change in bond length is attributable to the interaction of Zn, Pb, and Cu with O on the surface of kaolinite. In addition, after adsorption, the angle of [Zn(OH)]+, [Pb(OH)]+, and [Cu(OH)]+ with the surface of kaolinite became smaller. [Zn(OH)]+ and [Pb(OH)]+, and in particular the –OH group of [Pb(OH)]+, are more strongly attracted to the –OH group on the kaolinite alumina surface than [Cu(OH)]+. This may be because [Pb(OH)]+ is more likely to form H-bonds with –OH on the kaolinite surface. In an aqueous solution, the H-bonds between the surrounding water molecules and [Pb(OH)]+ are weaker. However, the –OH of [Cu(OH)]+ and those on the surface of kaolinite are not prone to H-bond formation and exhibit a slight repulsive effect.

#### Density of states analysis

**Figure 8:**
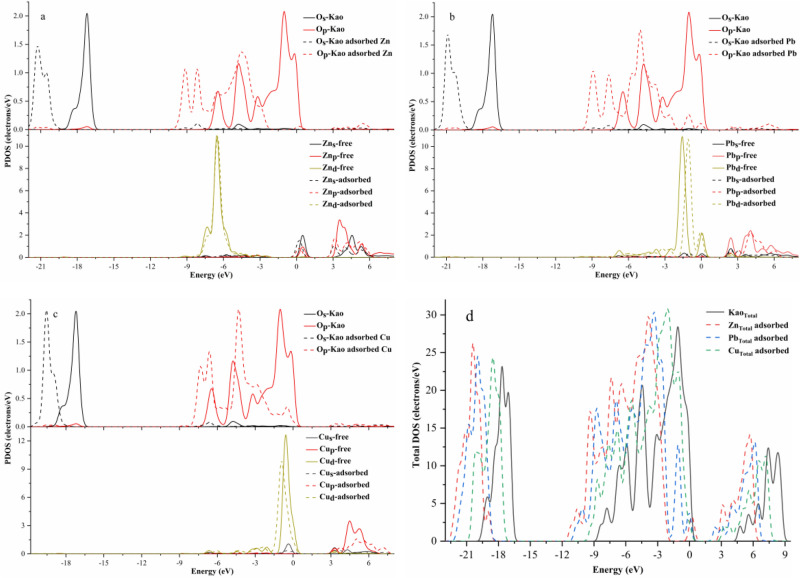
PDOS of O atoms on the surface of kaolinite (001) before (solid line) and after (dashed line) adsorption (**a**: [Zn(OH)]+, **b**: [Pb(OH)]+, **c**: [Cu(OH)]+, **d**: [TDOS)]+.

The bonding mechanism of Zn, Pb, and Cu on the Al–O surface of kaolinite is further discussed. The total density of states (TDOS) and partial density of states (PDOS) before and after the adsorption of Zn, Pb, and Cu in [Zn(OH)]+, [Pb(OH)]+, and [Cu(OH)]+, respectively, and on the surface O atoms of kaolinite Al–O were calculated. Before and after the adsorption of [Zn(OH)]+, [Pb(OH)]+ and [Cu(OH)]+ on the kaolinite (001) surface (Fig. 8d), the total orbital energy of kaolinite decreased by about 2.4, 1.9, and 0.57 eV, respectively, relative to the Fermi level. Simultaneously, the TDOS of [Zn(OH)]+, [Pb(OH)]+, and [Cu(OH)]+ increased after adsorption, indicating that [Zn(OH)]+, [Pb(OH)]+, and [Cu(OH)]+, respectively, were successfully adsorbed on the kaolinite surface, and the charge of the kaolinite orbitals was transferred and redistributed. Figure 8a shows the changes in the PDOS of O and Zn on the surface of kaolinite (001) before and after adsorption of [Zn(OH)]+. The combination of [Zn(OH)]+ and O on the surface of kaolinite has a significant influence on the O-s and O-p orbitals. The PDOS of the original O–p orbital was a narrow and high peak in the −1.5 to 0.68 eV range, but the energy band was shifted to the right after combination with [Zn(OH)]+ and its peak value decreased significantly. The two peaks of O–p at in the − 7.6 to − 10.3 eV range were clearly higher than those before adsorption, and the large height difference between two peaks in the − 3.75 to − 7.4 eV range was reduced. However, some new small peaks appeared in the 2.9–6.1 eV and − 0.1 to 0.6 eV ranges after adsorption, which is attributable to the electron transfer of [Zn(OH)]+. The Zn–s and Zn–p orbitals contributed electrons for the O-p and O-s orbitals in these regions. The Zn–s and Zn–p orbitals overlapped with the O–s and O–p orbitals, indicating that Zn formed a bond with O to create Zn–s–O–s and Zn–p–O–p hybridized orbitals. The PDOS of the O-s orbital in the − 19.5 to − 16.4 eV range, which was the main contributing orbital, changed significantly. The highest peak of O–s decreased by approximately 0.6 electrons/eV and the energy also decreased. The main reason for these changes is the electronic interaction between Zn in [Zn(OH)]+ and O atoms on the surface of kaolinite. Figure 8a shows that the PDOS of the Zn-d orbital was relatively strong, exhibiting a high and sharp distribution peak and indicating that this orbital fills more electrons. Moreover, the orbital change was not obvious after adsorption, because the Zn-d filled orbital hardly participated in bond formation when it reacted with other atoms. The PDOS of both the Zn–s and Zn–p orbitals decreased after [Zn(OH)]+ was adsorbed on kaolinite; their energy also decreased and the energy bands were close to the Fermi level, forming a more stable state. These results are consistent with the fact that an exothermic reaction has a negative adsorption energy.

For the [Pb(OH)]+ system (Fig. 8b), the overall energy of the energy band decreased after adsorption on kaolinite (001) surface, and the energy band of O atoms on the kaolinite surface also decreased. It is evident that the O-p orbital changes significantly after adsorption. Three new peaks appeared in the − 3 to 0.7 eV range and overlapped with those of the Pb-d orbital, indicating that Pb and O formed a stable Pb–O bond in the form of a Pb-d-O-p hybrid orbital. Additionally, in the 1.7–3 eV and 4.5–6.3 eV ranges, the Pb–s and Pb–p orbitals overlapped with the O-s and O-p orbitals of kaolinite, which is attributable to the close energy levels of the 6p orbital of Pb and 2p orbital of O. This proximity resulted in effective interaction between the two orbitals due to electron transfer, and finally the Pb–6p–O–2p bonding orbital was formed. The energy band resulting from the adsorption of Pb was shifted to the right compared with those resulting from the adsorption of Zn and Cu, which may be related to the direction of electron transfer. Due to its stable nature and high electron density. The main bonding mechanism involves interaction of the Pb–6p and O–2p orbitals to form bonding and anti-bonding orbitals, respectively, and further coupling of the Pb-6s orbital with the Pb–6p–O–2p anti-bonding orbital; the bonding and anti-bonding orbitals are filled simultaneously. Because the adsorption co-coordination structure is affected by the competitive coordination of H atoms, the surface O atoms of kaolinite and Pb atoms form weak bonds.

The change in PDOS after the adsorption of [Cu(OH)]+ on the aluminum oxide surface of kaolinite (001) is shown in Fig. 8c. The overall change in the PDOS of O for [Cu(OH)]+ was consistent with those for [Zn(OH)]+ and [Pb(OH)]+, and they all exhibited some leftward shift. The peak of the O-p orbital on the surface of kaolinite disappeared in the − 3 to − 3.5 eV range, but a new peak that showed obvious overlap with the Cu-d orbital appeared near the Fermi level (− 1.4 to 0.22 eV). The charge of the Cu-d orbital decreased after adsorption because of Cu(II) ions with unfilled 3d orbitals. The Cu–d and O–p orbitals contributed in the same energy range and formed Cu–d–O–p hybrid orbitals. Cu has sp^3^d^2^ hybridization and, thus, the hybrid orbitals can be further coupled with O–p orbitals. In addition, in the 3.75–6 eV range, the Cu–s and Cu–p orbitals overlapped with the O–s and O–p orbitals, indicating that Cu formed bonds with O on the Al–O surface of kaolinite (001). Upon adsorption on the Al–O surface of kaolinite, the molecules often undergo distortion of their geometric configuration to further reduce the energy of the system, which is consistent with the change in the Cu–OH bond length after the adsorption of Cu(OH) (Fig. 7e,f).

#### Electron density difference

Based on the previous results, because this work examines the interaction of [Zn(OH)]+, [Pb(OH)]+, and [Cu(OH)]+ with kaolinite, it is necessary to discuss the molecular electron distribution. The adsorption of [Zn(OH)]+, [Pb(OH)]+, and [Cu(OH]+) on kaolinite was modelled to discuss the direction of electron transfer during adsorption. The electron density difference is widely used in electron structure analysis because it can be used to study the electron movement and redistribution caused by the interaction between molecules or clusters and solid materials^45^. The electron density difference was calculated according to Eq. (5).

5 $$\Delta \delta = \sigma_{a/s} - \sigma_{a} - \sigma_{s}$$

where δ_a/s_ is the total charge of the adsorbed molecule and substrate and, δ_a_ and δ_s_ are the charge densities of adsorbed molecules and substrates, respectively. The adsorbed molecules in this study were [Zn(OH)]+, [Pb(OH)]+, and [Cu(OH)]+, and, the substrate was kaolinite.

**Figure 9:**
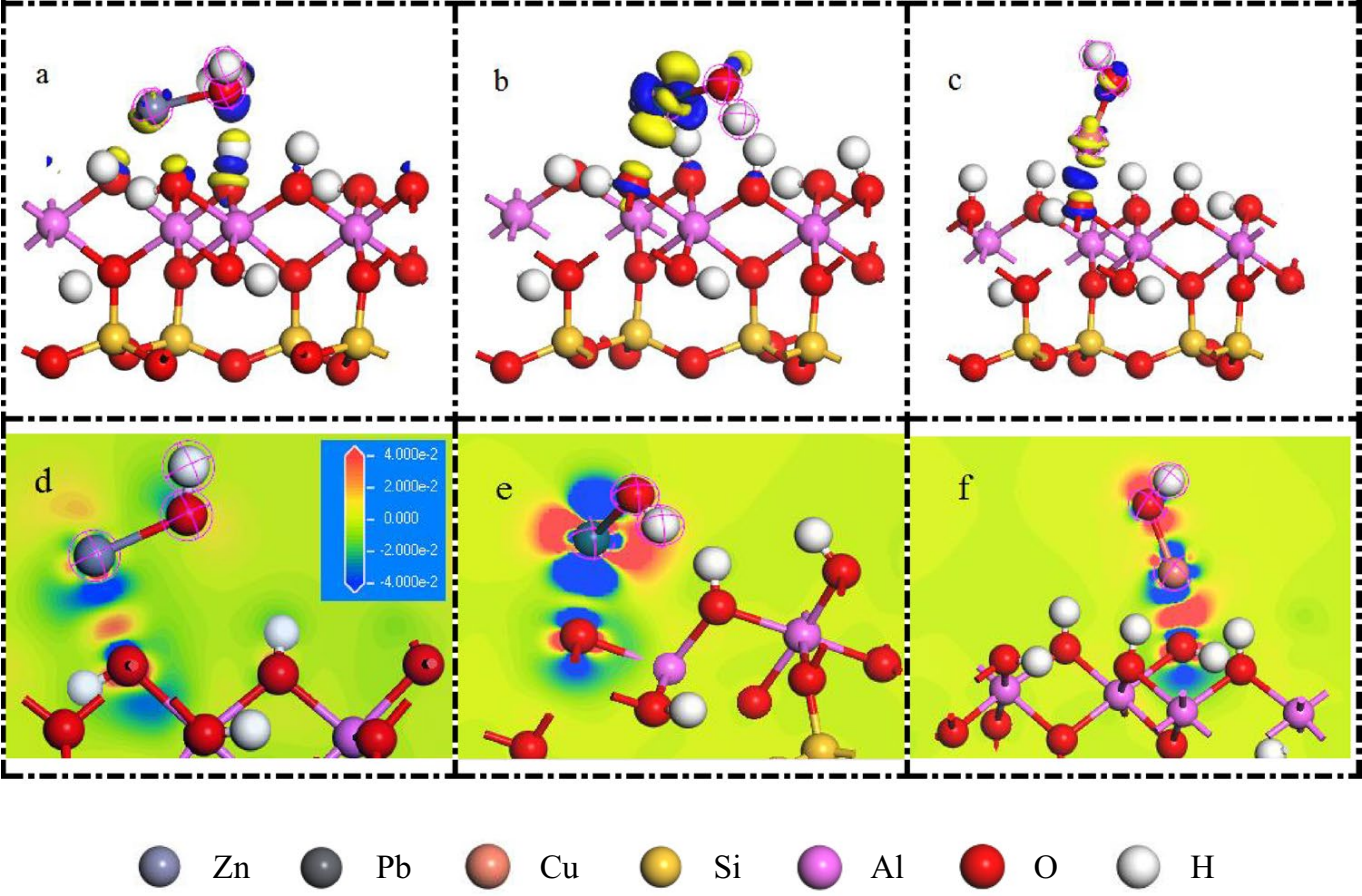
Electron density difference of [Zn(OH)]+ (**a**, **d**), [Pb(OH)]+ (**b**, **e**) and [Cu(OH)]+ (**c**, **f**).

The electron redistribution of [Zn(OH]+), [Pb(OH)]+, and [Cu(OH)]+ after adsorption on the surface of kaolinite (001) is shown in Fig. 9. When the isosurface is 0.05 e A^−3^, the electron interactions between [Zn(OH)]+, [Pb(OH)]+, and [Cu(OH)]+ are obvious. For the [Zn(OH)]+ system, although Zn was placed at the O_u_H position, it clearly has stronger interactions with the nearby O_l_H based on the electron density difference shown in Fig. 9a, d. At the same isosurface, the interaction of [Zn(OH)]+ with the O atoms on the kaolinite surface was weaker than those of [Pb(OH)]+ and [Cu(OH)]+.This result is consistent with the calculated adsorption energy of − 0.49 eV. In addition, electron transfer between [Zn(OH)]+ and the other O atoms around the kaolinite surface is more obvious (Fig. 9d), indicating that [Zn(OH)]+ is more likely to undergo multi-tooth adsorption. Interestingly, for the adsorption of [Zn(OH)]+ and [Cu(OH)]+, the O atoms on the kaolinite surface act as electron acceptors and the metal ions as electron donors. However, for the [Pb(OH)]+ system, the opposite characteristics are observed. This result is consistent with the DOS after [Pb(OH)]+ adsorbed that the orbital energy increased. The specific reasons for this behavior warrant further exploration.

## Conclusions

In the present study, the adsorption behavior of original kaolinite for Zn(II), Pb(II), and Cu(II) was discussed under different conditions. The adsorption isotherms agreed with the Langmuir monolayer adsorption model, and the maximum adsorption capacities of Zn(II), Pb(II), and Cu(II) on kaolinite were 15.515, 61.523, and 44.659 mg/g, respectively, at pH 5.0 and 298 K. The XRD, FI-TR, and SEM–EDS results showed that metal ions cannot enter the interlayers easily and are mainly absorbed on the kaolinite surface. The special lamellar spatial structure of kaolinite and high content of O-containing functional groups on the kaolinite surface makes it easy to form chemical interactions and physical synergies related to pollutants on the mineral surface, such as chemical bonding through complexation and hydroxide formation through electrostatic attraction. Based on the periodic model of kaolinite and DFT calculations, the changes in the microstructure and electronic structure of original kaolinite after adsorption were studied. The theoretical calculation results are in agree with the experimental results. The microscopic views show that the metal ions form new hybrid orbitals with the O atoms from the –OH groups on the kaolinite surface, resulting in charge transfers and structural changes. The direction of charge transfer for [Pb(OH)]+ was opposite those for [Zn(OH)]+ and [Cu(OH)]+. Moreover, [Zn(OH)]+ was more likely to form polydentate complexes with –OH groups on the kaolinite surface. This work explains the adsorption mechanisms of Zn, Pb, and Cu ions on the kaolinite surface and is expected to further elucidate the microscopic mechanisms involved at the interface between metal ions and clay minerals. This study provides new insights into the mechanism of interaction between kaolinite and metal ions, and provides new directions for the design of metal ion adsorption materials, such as intercalation to increase the intervals between layers and modification to increase the amount of charge transfer. This exploration will play an important role in the remediation of heavy metal pollution through the modified clay minerals.

## Data Availability

The datasets used and analysed during the current study available from the corresponding author on reasonable request.
